# Engineered Human Meniscus in Modeling Sex Differences of Knee Osteoarthritis *in Vitro*

**DOI:** 10.3389/fbioe.2022.823679

**Published:** 2022-02-15

**Authors:** Zhiyao Ma, David Xinzheyang Li, Melanie Kunze, Aillette Mulet-Sierra, Lindsey Westover, Adetola B. Adesida

**Affiliations:** ^1^ Department of Surgery, Divisions of Orthopaedic Surgery, Surgical Research and Otolaryngology-Head and Neck Surgery, Faculty of Medicine and Dentistry, University of Alberta, Edmonton, AB, Canada; ^2^ Department of Civil and Environmental Engineering, University of Alberta, Edmonton, AB, Canada; ^3^ Department of Mechanical Engineering, University of Alberta, Edmonton, AB, Canada

**Keywords:** disease modelling, human-engineered meniscus, mechanical stimulation, simulated microgravity, cyclic hydrostatic pressure

## Abstract

**Background:** Osteoarthritis (OA) primarily affects mechanical load-bearing joints. The knee joint is the most impacted by OA. Knee OA (KOA) occurs in almost all demographic groups, but the prevalence and severity are disproportionately higher in females. The molecular mechanism underlying the pathogenesis and progression of KOA is unknown. The molecular basis of biological sex matters of KOA is not fully understood. Mechanical stimulation plays a vital role in modulating OA-related responses of load-bearing tissues. Mechanical unloading by simulated microgravity (SMG) induced OA-like gene expression in engineered cartilage, while mechanical loading by cyclic hydrostatic pressure (CHP), on the other hand, exerted a pro-chondrogenic effect. This study aimed to evaluate the effects of mechanical loading and unloading *via* CHP and SMG, respectively, on the OA-related profile changes of engineered meniscus tissues and explore biological sex-related differences.

**Methods:** Tissue-engineered menisci were made from female and male meniscus fibrochondrocytes (MFCs) under static conditions of normal gravity in chondrogenic media and subjected to SMG and CHP culture. Constructs were assayed *via* histology, immunofluorescence, GAG/DNA assays, RNA sequencing, and testing of mechanical properties.

**Results:** The mRNA expression of *ACAN* and *COL2A1*, was upregulated by CHP but downregulated by SMG. *COL10A1*, a marker for chondrocyte hypertrophy, was downregulated by CHP compared to SMG. Furthermore, CHP increased GAG/DNA levels and wet weight in both female and male donors, but only significantly in females. From the transcriptomics, CHP and SMG significantly modulated genes related to the ossification, regulation of ossification, extracellular matrix, and angiogenesis Gene Ontology (GO) terms. A clear difference in fold-change magnitude and direction was seen between the two treatments for many of the genes. Furthermore, differences in fold-change magnitudes were seen between male and female donors within each treatment. SMG and CHP also significantly modulated genes in OA-related KEGG pathways, such as mineral absorption, Wnt signalling pathway, and HIF-1 signalling pathway.

**Conclusion:** Engineered menisci responded to CHP and SMG in a sex-dependent manner. SMG may induce an OA-like profile, while CHP promotes chondrogenesis. The combination of SMG and CHP could serve as a model to study the early molecular events of KOA and potential drug-targetable pathways.

## Introduction

Osteoarthritis (OA) is the most common form of degenerative disease and primarily affects loading-bearing joints, with the knee joint being the most prevalent (Nicolella et al., 2012; Boyan et al., 2013a). A hallmark feature of knee osteoarthritis (KOA) is the atrophy of articular cartilage. Surrounding joint tissues, including the menisci, will also undergo breakdown (Sun et al., 2010; Blackburn et al., 2016). These abnormal changes can lead to rapid loss of the knee joint’s function and mobility, making KOA a leading cause of physical disability (Murray et al., 2012). Although KOA occurs in almost all demographic groups, the prevalence and severity of KOA increases with age and is disproportionately higher in females than males (Badley and Kasman, 2004; Breedveld, 2004; O’Connor, 2007; Boyan et al., 2012; Pan et al., 2016). It has been reported by the World Health Organization that 9.6% of males and 18% of females above the age of 60 years have symptomatic OA (Osteoarthritis, 2014).

The molecular mechanisms and cellular events underlying the pathogenesis and progression of KOA are not well understood. As well, the molecular basis of biological sex matters has not been previously investigated. Currently, there is no consensus model to reflect the pathophysiology of KOA holistically. However, the molecular and cellular characteristics of KOA resemble the hypertrophic differentiation of chondrocytes as they progress to the bone during endochondral ossification (Dreier, 2010). Conveniently, this process includes the upregulation of hypertrophic markers *COL10A1* and *MMP13*, which can be indicators for the KOA phenotype (Aigner et al., 1993; D’Angelo et al., 2000). Healthy chondrocytes, on the other hand, resist hypertrophic differentiation and lack expression of these hypertrophic markers (Leijten et al., 2012). Given the disproportionate incidence of KOA in females as compared to males, we reasonably expect the cellular and molecular characteristics of KOA to show sex-dependent differences.

Mechanical stimulation was reported by an abundance of studies to play a critical role in modulating OA-related responses of loading-bearing tissues. Applying mechanical loading to joints through regular exercise is essential to maintaining healthy cartilage and preventing breakdown from prolonged disuse (Bader et al., 20112011). Cyclic hydrostatic pressure (CHP) as a loading modality mimics physiological loading patterns and can be easily recreated *in-vitro* using specialized bioreactors (Elder and Athanasiou, 2009; Mellor et al., 2017). Studies have shown that CHP applied to engineered tissue constructs has induced a mostly pro-chondrogenic effect. For example, Zellner et al. applied dynamic hydrostatic pressure (cyclic at 1 Hz for 4 h per day, 0.55–5.03 MPa) for 7 days to cellular aggregates generated from inner and outer meniscus fibrochondrocytes (MFCs) (Zellner et al., 2015). After 14 additional days of static culture, aggregates loaded initially for the 7 days showed immunohistochemically enhanced chondrogenesis compared to unloaded controls (Zellner et al., 2015). Further, Gunja et al. applied dynamic hydrostatic pressure (0.1 Hz for 1 h every 3 days, 10 MPa) for 28 days to engineered tissue constructs using MFCs with added TGF-β1 growth factors (Gunja et al., 2009). Loaded tissue constructs with growth factors showed additive and synergistic increases in collagen deposition (approximately 2.5-fold), GAG deposition (2-fold), and enhanced compressive properties compared to unloaded controls without growth factors (Gunja et al., 2009).

Mechanical unloading of joints from long-term immobilization has been shown to induce cartilage atrophy that resembles characteristics of KOA. In a case study by Souza et al., joint immobilization of healthy individuals without prior history of OA resulted in magnetic resonance imaging (MRI) parameters of their knee articular cartilage that resemble KOA (Souza et al., 2012). When returning to standard weight-bearing, the MRI parameters for the joints were restored to baseline values consistent with healthy articular cartilage (Souza et al., 2012). Mechanical unloading has also been modelled by simulated microgravity (SMG) using rotating wall vessel bioreactors (Yu et al., 2011; Jin et al., 2013; Mayer-Wagner et al., 2014; Mellor et al., 2014; Mellor et al., 2017). Mayer-Wagner et al. applied simulated microgravity for 21 days to human mesenchymal stem cell (hMSC) pellets and found a decrease in histological staining of proteoglycans and collagen type-II compared to normal gravity controls (Mayer-Wagner et al., 2014). SMG pellets also showed a lower *COL2A1*/*COL10A1* expression ratio suggesting that mechanical unloading *via* SMG reduced the chondrogenic differentiation of hMSCs (Mayer-Wagner et al., 2014). Finally, in a comparative study, CHP-loaded pellets from human adipose-derived stem cells showed increased expression of *ACAN*, *SOX9*, and *COL2A1*, and a 3-fold increase in GAG productions compared to unloaded SMG groups (Mellor et al., 2017). However, none of the above studies investigated the sex-dependent differences in the magnitude of differential modulation by mechanical loading and unloading *via* CHP and SMG, respectively.

A recent definition of OA from the Osteoarthritis Research Society International includes the menisci of the knee joint as a tissue undergoing breakdown and abnormal changes from the disease (Blackburn et al., 2016). Knee menisci undergoing OA has also been shown to have similar characteristics as knee articular cartilage undergoing OA, such as focal calcification and the increased expression of hypertrophic markers *COL10A1* and *MMP13* (Sun et al., 2010; Kiraly et al., 2017). This suggests that MFC may play an active role in the pathogenesis of KOA alongside knee articular chondrocytes, making MFC a reasonable cell option to model OA *in-vitro*.

Taken together, the goal of this study was to evaluate the effects of mechanical loading and unloading *via* CHP and SMG, respectively, and determine sex-dependent differences in the modulation of OA-related characteristics in bioengineered human meniscus tissues. This would serve as an OA disease model *in-vitro* to determine the cellular and molecular profiles responsible for the sex-dependent incidence of the disease.

## Materials and Methods

**FIGURE 1:**
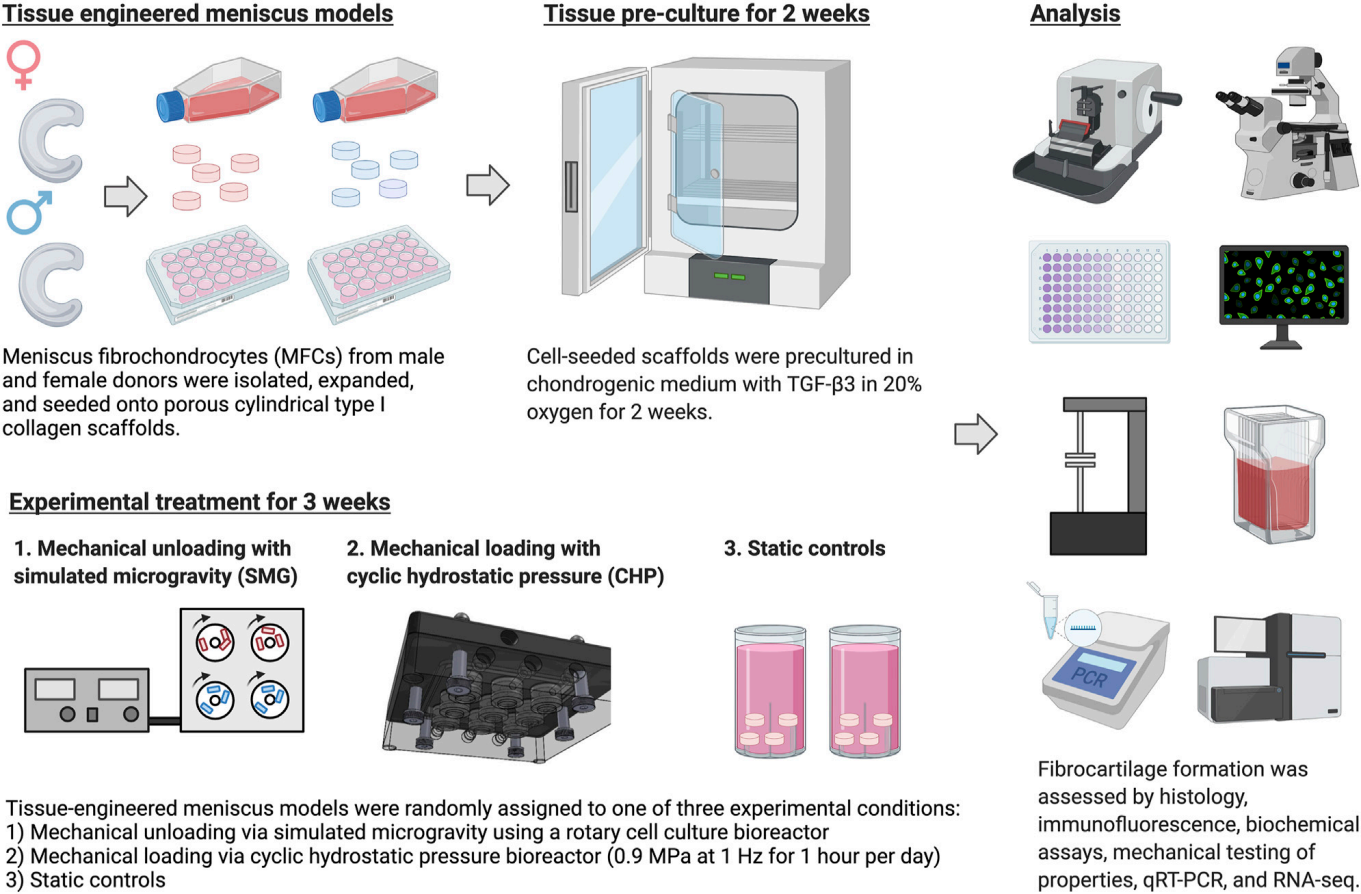
Experiment outlines. Created with Biorender.com (2021).

The experiment is outlined in Figure 1. Most culture methods and assays were performed identically to those described in previous work (Liang et al., 2017; Szojka et al., 2021a; Szojka et al., 2021b).

### Ethics Statement

Human non-osteoarthritic inner meniscus samples were collected from patients undergoing partial meniscectomies at the University of Alberta Hospital and Grey Nuns Community Hospital in Edmonton. The ethics of this study was approved by the Health Research Ethics Board of the University of Alberta. Non-identifying donor information is listed in Table 1.

**TABLE 1:** Non-identifying donor information.

Sex	Donor number	Age	Population doubling (PD)
Female	F1	33	2.687
	F2	44	2.380
	F3	30	2.774
	F4	28	3.860
Male	M1	19	3.349
	M2	45	2.699
	M3	22	3.247
	M4	35	2.149

### Cell and Tissue Culture

Meniscus fibrochondrocytes (MFCs) were isolated from inner meniscus tissue samples by type II collagenase (0.15% w/v of 300 units/mg; Worthington) mediated digestion, followed by 48 h recovery. After recovery, cells were replated in tissue culture flasks at the density of 10^4^ cells/cm^2^ and expanded for 1 week in high glucose Dulbecco’s modified Eagle’s medium (HG-DMEM) supplemented with 10% v/v heat-inactivated fetal bovine serum (FBS), 10 mM 4-(2-hydroxyethyl)-1-piperazineethanesulfonic acid (HEPES), 100 U/mL penicillin, 100 μg/ml streptomycin and 2 mM L-glutamine (PSG; Life Technologies, ON, Canada), 5 ng/ml of FGF-2 (Neuromics, MH, United States, catalogue #: PR80001) and 1 ng/ml of TGF-β1 (ProSpec, catalogue #: CYT-716) for 1 week

Expanded MFCs were resuspended in defined chondrogenic medium (HG-DMEM supplemented with HEPES, PSG, ITS +1 premix (Corning, Discovery Labware, Inc, MA, United States), 125 μg/ml of human serum albumin, 100 nM of dexamethasone, 365 μg/ml ascorbic acid 2-phosphate, 40 μg/ml of l-proline, and 10 ng/ml of TGF-β3) and seeded onto bovine type I collagen scaffolds (dimensions: 6 mm diameter, 3.5 mm height; Integra LifeSciences, NJ, United States) at the density of 5×10^6^ cells/cm^3^. The cell-containing constructs were precultured statically in standard 24-well plates with 2.5 ml/construct of the chondrogenic medium described above for 2 weeks; media changes occurred once per week.

### Mechanical Stimulation

After the 2-weeks preculture, tissue constructs were randomly assigned to a mechanical stimulation group. For the static control group, constructs were placed in a tissue culture tube (Sarstedt, Germany). For the mechanical unloading group, a commercially available bioreactor (RCCS-4; Synthecon Inc.) was used to culture tissue constructs in a simulated microgravity (SMG) environment. The rotation speed was adjusted over time to maintain constructs in suspension (30 rpm from day 1–2; 34 rpm from day 3–7; 37 rpm from day 8–13; 40 rpm from day 14–21). For the mechanical loading group, cyclic hydrostatic pressure (CHP) was applied to tissue constructs using a MechanoCulture TR (CellScale, Canada). Constructs were loaded 1 h per day and daily with 0.9 MPa cyclic hydrostatic pressure at the frequency of 1 Hz. When not loaded, tissue constructs were cultured in 6-well plates under static conditions. All experimental groups were cultured with chondrogenic medium with supplemented TGF-β3 growth factor, and the volume of medium per tissue construct (approximately 6.5 ml per tissue construct per week) was equivalent among the different groups. The mechanical stimulation was applied for 3 weeks, and medium change was performed once a week. At the end of the 3-weeks treatment, the CHP group was allowed 30 min of rest following the last loading event for gene expression changes to occur. Tissue replicates for RNA extraction were placed in TRIzol reagent and frozen at −80°C. Constructs from SMG and static control groups were harvested at approximately the same time.

### Histology, Immunofluorescence, and Biochemical Analysis

The wet weight of tissue constructs (n = 5–8 replicates) intended for histology and biochemical analysis was recorded at the end of the experiment. Constructs (n = 2 replicates, only one replicate is presented) were then fixed in 1 ml of 10% v/v buffered formalin (Fisher Scientific, MA, United States) overnight at 4°C, dehydrated, embedded in paraffin wax, and sectioned at 5 µm thickness. Tissue sections approximately from the middle region of the constructs were stained with Safranin-O (Sigma-Aldrich, United States, #S2255-25G), Fast Green FGF (Sigma-Aldrich, United States, #F7258-25G), and Haematoxylin (Sigma-Aldrich, United States, #MHS32-1L) for histological examination of cell morphology and extracellular matrix deposition. Briefly, for immunofluorescence staining, tissue sections were labelled with primary antibody against human type I, type II, and type X collagen (1:200 dilution of anti-human rabbit type I collagen, Cedarlane, Canada, #CL50111AP-1; 1:200 dilution of mouse anti-human type II collagen, Developmental Studies Hybridoma Bank, United States, #II-II6B3; 1:100 dilution of rabbit anti-human type X collagen, Abcam, UK, #ab58632) and incubated overnight at 4°C (Anderson-Baron et al., 2021). On the next day, the secondary antibody (1:200 dilution of goat anti-rabbit, Abcam, UK, #ab150080; 1:200 dilution of goat anti-mouse, Abcam, UK, #ab150117) and DAPI (Cedarlane, Canada) was applied to visualize the stained components.

Biochemical assays quantified the total content of glycosaminoglycan (GAG) and DNA. Tissue constructs (n = 4 replicates for donors F1-3 and M1-3; n = 3 replicates for donors F4 and M4) were digested overnight with proteinase K (Sigma-Aldrich, United States, #P2308) at 56°C. GAG content was measured with a 1,9-dimethyl methylene blue assay (DMMB, Sigma-Aldrich, United States, #341088). Chondroitin sulphate (Sigma-Aldrich, United States, #C8529) was used to generate the standard curve. DNA content was measured with a CyQuant cell proliferation assay kit (ThermoFisher Scientific, United States, #C7026) with different dilutions of supplied bacteriophage λ DNA as the standard.

### Mechanical Property Assessment

Detailed sequence of the mechanical testing protocol is included in Supplementary Material S3. A stepwise stress relaxation test (Supplementary Figure S1) was used to assess the mechanical properties of tissue constructs (n = 2 replicates for donor F1-3 and M1-3) with the BioDynamic 5210 system (TA Instruments, United States). The cross-section areas of tissue constructs were measured before mechanical tests. For the test, constructs were placed between two platens and the initial height was determined by bringing tissue to near contact with platens. Constructs were first preconditioned by 15 cycles of sine wave dynamic loading with the amplitude of 5% tissue height at the frequency of 1 Hz. The following stress relaxation test consisted of 3 incremental strain steps. In the first two steps, the constructs were subjected to a 10% strain ramp at the rate of 50% strain/sec followed by 5 min relaxation under constant strain. In the third step, the relaxation time was adjusted to 10 min. All tested constructs were able to reach equilibrium within the given relaxation period. Force was recorded as a function of time, and stress was calculated by normalizing force to construct’s cross-section area. The peak modulus was calculated by dividing the maximum stress measured immediately after each strain increment by the strain increment. The strain was applied in 10% increments up to a maximum of 30% strain.

### RNA Extraction, qPCR, and Next-Generation Sequencing

Tissue constructs (n = 2 for donors F1-3 and M1-3; n = 3 replicates for donors F4 and M4) intended for transcriptome analysis were preserved in Trizol (Life Technologies, United States) immediately upon harvesting and stored at −80°C until RNA extraction. RNA was extracted and purified from ground constructs using PuroSPIN Total DNA Purification KIT (Luna Nanotech, Canada) following the manufacturer’s protocol. RNA was reversely transcribed into cDNA, and genes of interest were amplified by quantitative real-time polymerase chain reaction (RT-qPCR) using specific primers (Supplementary Table S1). The expression level of genes of interest was normalized to chosen housekeeping genes (i.e., *B-actin*, *B2M*, and *YWHAZ*) based on the coefficient of variation (CV) and M-value as measures of reference gene stability (Hellemans et al., 2007), and the data was presented using the 2^-∆∆CT^ method (Livak and Schmittgen, 2001; Schmittgen and Livak, 2008). Next-generation RNA-sequencing was performed on the Illumina NextSeq 500 platform with paired-end 42 bp × 42 bp reads, and FastQ files were obtained for further bioinformatics analysis.

### Bioinformatics

Next-generation sequencing data were analyzed with Partek^®^ Flow^®^ software (Version 10.0.21.0302, Copyright^©^ 2021, Partek Inc, St. Louis, MO, United States). Raw input reads were first trimmed from the 3′ end to achieve a quality score beyond 20 and then aligned to the reference human genome hg38 using the STAR 2.7.3a aligner. Aligned data were quantified to a transcript model (hg38-RefSeq Transcripts 94–2020–05–01) using the Partek E/M algorithm. Genes with maximum read counts below 50 were filtered out to reduce noise. Quantified and filtered reads were normalized in sequential order using the Add: 1.0, TMM, and Log 2.0 methods. Statistical analysis was performed using analysis of variance (ANOVA) for biological sex and treatment. Within each sex, the donors were assigned as a random variable. Differentially expressed genes (DEGs) for each comparison were determined by *p*-values, adjusted *p*-values (*q*-values), and fold change (FC). Gene ontology enrichment, pathway enrichment, and the visualization of DEGs using Venn diagrams were all conducted in Partek.

### Statistical analysis

Statistical analyses were performed in Prism 9 (GraphPad) and Partek^®^ Flow^®^ software. The statistical test used, and *p*-values and *q*-values are indicated in the respective figure legends. For analysis in Figure 3B, paired *t*-test were used between SMG and CHP groups within each sex and unpaired *t*-test were used between female and male groups within each mechanical treatment. For analysis in Figure 4A, a repeated measurement one-way ANOVA with Geisser-Greenhouse correction was used to compare mechanical treatment groups within each sex and a Tukey’s multiple comparison test was used to find the adjusted *p*-value between each comparison. Within each mechanical treatment, unpaired t-tests between female and male groups were used. For the male cohort, no statistical analysis was conducted for the SMG group in contraction due to limited data points. For analysis in Figure 4B, repeated measurement one-way ANOVA with Geisser-Greenhouse correction was used to compare different strain levels within each mechanical treatment group and a Tukey’s multiple comparison test was used to find the adjusted *p*-value between each comparison. Within each strain level, paired t-tests were used to compare CHP and SMG groups. For the male cohort, no statistical analysis was conducted for the SMG group due to limited data points.

## Results

### Dataset Overview

Transcriptome analysis included the expression profiles of 8 donors (4 females and 4 males), each individually exposed to static, mechanical loading (CHP), and mechanical unloading (SMG) conditions. After preprocessing as described in the methods, 13,361 genes were preserved for downstream analysis.

### Transcriptome Profiles of the Engineered Meniscus to CHP and SMG

**FIGURE 2:**
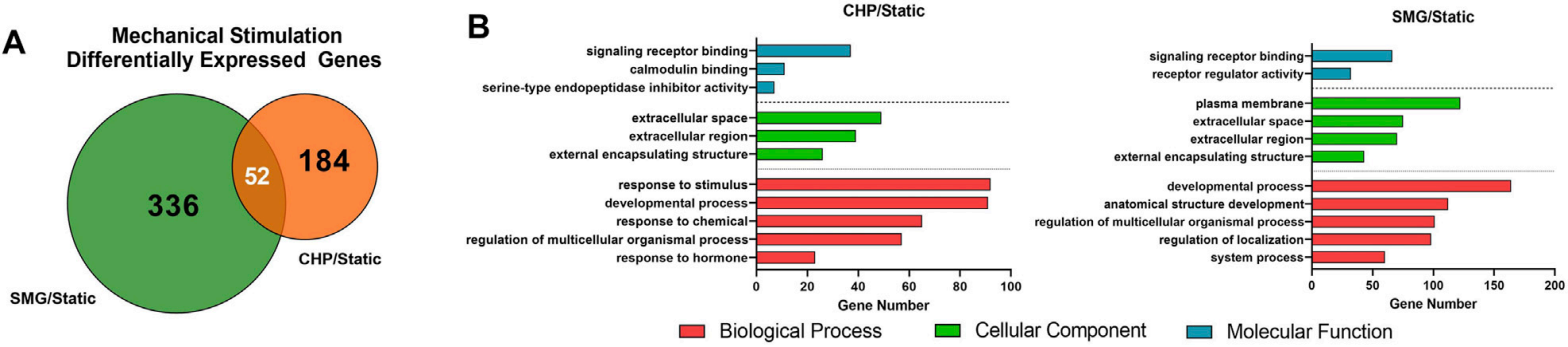
Effect of CHP and SMG on the transcriptome profile alteration of engineered meniscus tissues. **(A)** Overlap of differentially expressed genes (DEGs) by CHP and SMG. **(B)** Top non-redundant Gene Ontology (GO) terms enriched by DEGs of CHP and SMG. DEGs were identified based on all 8 donors. Top significant enriched GO terms were selected by *p*-value and plotted to the number of genes included in each term.

We first analyzed the overall effect of mechanical loading and unloading on the transcriptome profiles of all donors combined. Summarized results are shown in Figure 2. Fold-change of gene expression levels in the CHP and SMG groups were calculated by normalizing to its corresponding static group. Differentially expressed genes (DEGs) were defined as genes with expression fold-change over 2 and *q*-value less than 0.05. Mechanical loading from CHP significantly modulated 236 genes, while mechanical unloading from SMG significantly modulated 388 genes. The overlay of DEGs between the two mechanical stimulation groups showed only a small proportion of common DEGs (52 genes), whereas the majority of DEGs were uniquely modulated by CHP (184 genes) and SMG (336 genes) (Figure 2A). These results indicated that CHP and SMG distinctly modulate the transcriptome profile of donors in this study.

The most significant Gene Ontology (GO) terms enriched by the DEGs for CHP and SMG were examined next (Figure 2B). Although the top 3 most significantly enriched Gene Ontology (GO) in biological components were identical for CHP and SMG (“extracellular space,” “extracellular region,” and “extracellular matrix”), the included gene expression profiles were different between the treatment groups. The top 20 genes with the highest absolute fold change participating in the ECM relevant activities in CHP and SMG groups are listed in Table 2. For CHP, most of the genes are signalling molecules or proteins associated with ECM structure remodelling. For SMG, many of the strongly regulated genes played a more general role, such as various growth factors coding genes: *IGFBP1*, *TGFA*, and *NGF*. Among the top regulated genes, only *NETO1* and *OLFML2A* were common between CHP and SMG; these two genes were upregulated in both treatment groups.

**TABLE 2:** Top 20 genes with the highest absolute fold change participating in the ECM relevant activities in CHP and SMG groups as compared to static controls.

*Gene*	Description	*p*-value: CHP vs static	Fold change: CHP vs static
*EREG*	Epiregulin	1.20E-06	19.10
*NET O 1*	Neuropilin And Tolloid Like 1	2.53E-08	7.98
*MMP3*	Matrix metallopeptidase 3	1.22E-06	6.47
*MMP10*	Matrix metallopeptidase 10	1.14E-03	4.54
*SBSPON*	Somatomedin B And Thrombospondin Type 1 Domain Containing	7.88E-04	3.97
*CCL2*	C-C Motif Chemokine Ligand 2	8.18E-07	3.62
*UCN2*	Urocortin 2	5.86E-05	3.61
*PLA2G2A*	Phospholipase A2 Group IIA	7.00E-04	3.60
*OLFML2A*	Olfactomedin Like 2A	1.15E-03	3.45
*CXCL13*	C-X-C Motif Chemokine Ligand 13	1.00E-03	3.40
*SFRP2*	Secreted Frizzled Related Protein 2	1.17E-05	−4.07
*ADM*	Adrenomedullin	1.34E-06	−4.49
*CPXM1*	Carboxypeptidase X, M14 Family Member 1	1.95E-04	−4.53
*SELENOP*	Selenoprotein P	3.90E-03	−5.82
*FAM20A*	FAM20A Golgi Associated Secretory Pathway pseudokinase	2.24E-03	−5.92
*MNDA*	Myeloid Cell Nuclear Differentiation Antigen	4.02E-03	−5.99
*SCUBE1*	Signal Peptide, CUB Domain And EGF Like Domain Containing 1	3.28E-03	−7.06
*SFRP4*	Secreted Frizzled Related Protein 4	6.83E-04	−8.17
*ODAPH*	Odontogenesis Associated Phosphoprotein	3.30E-03	−9.07
*APOE*	Apolipoprotein E	3.30E-07	−14.78

*Gene*	Description	*p*-value: SMG vs static	Fold change: SMG vs static
*IGFBP1*	Insulin Like Growth Factor Binding Protein 1	5.49E-04	15.94
*OLFML2A*	Olfactomedin Like 2A	2.34E-05	6.34
*NET O 1*	Neuropilin And Tolloid Like 1	1.26E-06	5.00
*ADAMTS14*	ADAM metallopeptidase With Thrombospondin Type 1 Motif 14	8.34E-04	3.72
*PCSK9*	Proprotein convertase Subtilisin/Kexin Type 9	2.20E-03	3.48
*VSTM2L*	V-Set And Transmembrane Domain Containing 2 Like	3.03E-04	3.47
*BMPER*	BMP Binding Endothelial Regulator	1.91E-04	3.45
*NTM*	Neurotrimin	1.30E-04	3.40
*HMOX1*	Heme oxygenase 1	5.06E-08	3.38
*CAPG*	Capping Actin Protein, Gelsolin Like	2.94E-07	3.22
*LEP*	Leptin	8.50E-04	−4.81
*R3HDML*	R3H Domain Containing Like	8.67E-04	−4.93
*APLN*	Apelin	8.68E-07	−4.94
*STC1*	Stanniocalcin 1	8.99E-05	−5.41
*NGF*	Nerve Growth Factor	7.68E-05	−5.42
*TGFA*	Transforming Growth Factor Alpha	8.23E-04	−5.47
*VEGFA*	Vascular Endothelial Growth Factor A	6.33E-06	−5.68
*DSCAML1*	DS Cell Adhesion Molecule Like 1	3.09E-03	−6.17
*ADAMTSL2*	ADAMTS Like 2	4.74E-04	−7.23
*PDE4C*	Phosphodiesterase 4C	2.48E-04	−8.73

### Sex-Dependent Response of Engineered Meniscus to CHP and SMG

**FIGURE 3:**
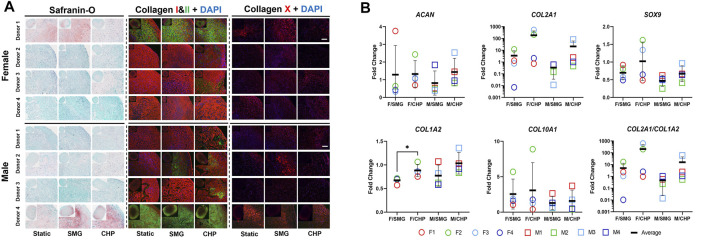
Effect of CHP and SMG on the chondrogenic and hypertrophic differentiation potential of female and male donors. **(A)** Histological and immunofluorescent staining analysis. **(B)** Regulation of selected genes expression level. Gene expression level of individual samples were measured by RT-qPCR. Fold change of expression level was calculated by normalizing expression level in the CHP or SMG group to its corresponding static group. Scale bar: 100 μm *Represents *p* < 0.05.

Next, we sought to explore the sex-dependent differences in the engineered meniscus responses to CHP and SMG. Therefore, we separated donors into female and male cohorts and evaluated several factors involved in normal cartilage physiology and OA-related alterations. The GAG and type II collagen content are often used to characterize the degree of chondrogenesis for cartilage tissues, and higher content is linked to higher chondrogenic capacity. Type X collagen, on the other hand, is a hypertrophic marker that suggests an OA-like phenotype. The histological staining for GAG by Safranin-O and immunofluorescence labelled type II collagen (Figure 3A) showed highly variable chondrogenic capacities within the female and male cohorts in the baseline static control group. Regardless, tissue constructs exposed to CHP and SMG showed an increase in type II collagen content, with CHP having a generally more pronounced effect. Type X collagen staining intensity was also modulated by mechanical stimulations compared to baseline. A clear increase in type X collagen content was observed in the SMG group, while the CHP group showed comparable type X collagen intensity as the baseline.

To further pursue sex matters in responses to CHP and SMG, the gene expression of selected markers was examined by RT-qPCR (Figure 3B). In addition to *COL1A2*, *COL2A1*, and *COL10A1*, the transcription factor *SOX9* and cartilage-specific proteoglycan core protein *ACAN* were also quantified. The observed increase of type II collagen level in the CHP group was confirmed quantitatively by gene expression results. The average *COL2A1* expression level was upregulated 215.9-fold in the CHP group for the female cohort compared to a 21.4-fold increase in CHP for the male cohort. For *ACAN*, *SOX9*, and *COL1A2*, the CHP group had a higher average fold-change than the SMG group, but the differences between the sexes were not significant. For *COL10A1*, the average fold-change of expression level was comparable between the CHP and SMG groups for both females and males. However, when taking individual female donors into account, only female donor 2 showed a significant increase in *COL10A1* expression level in CHP (8.9-fold) compared to SMG (5.7-fold) group. Generally, CHP, as compared to SMG, reduced *COL10A1* expression level for the female cohort. The RT-qPCR data were consistent with the histological observations, providing additional evidence on the cellular and molecular influence of CHP and SMG. The RT-qPCR data also agreed with the RNA-sequencing data, thereby providing additional validation for the observed trends (Supplementary Figure S2).

**FIGURE 4:**
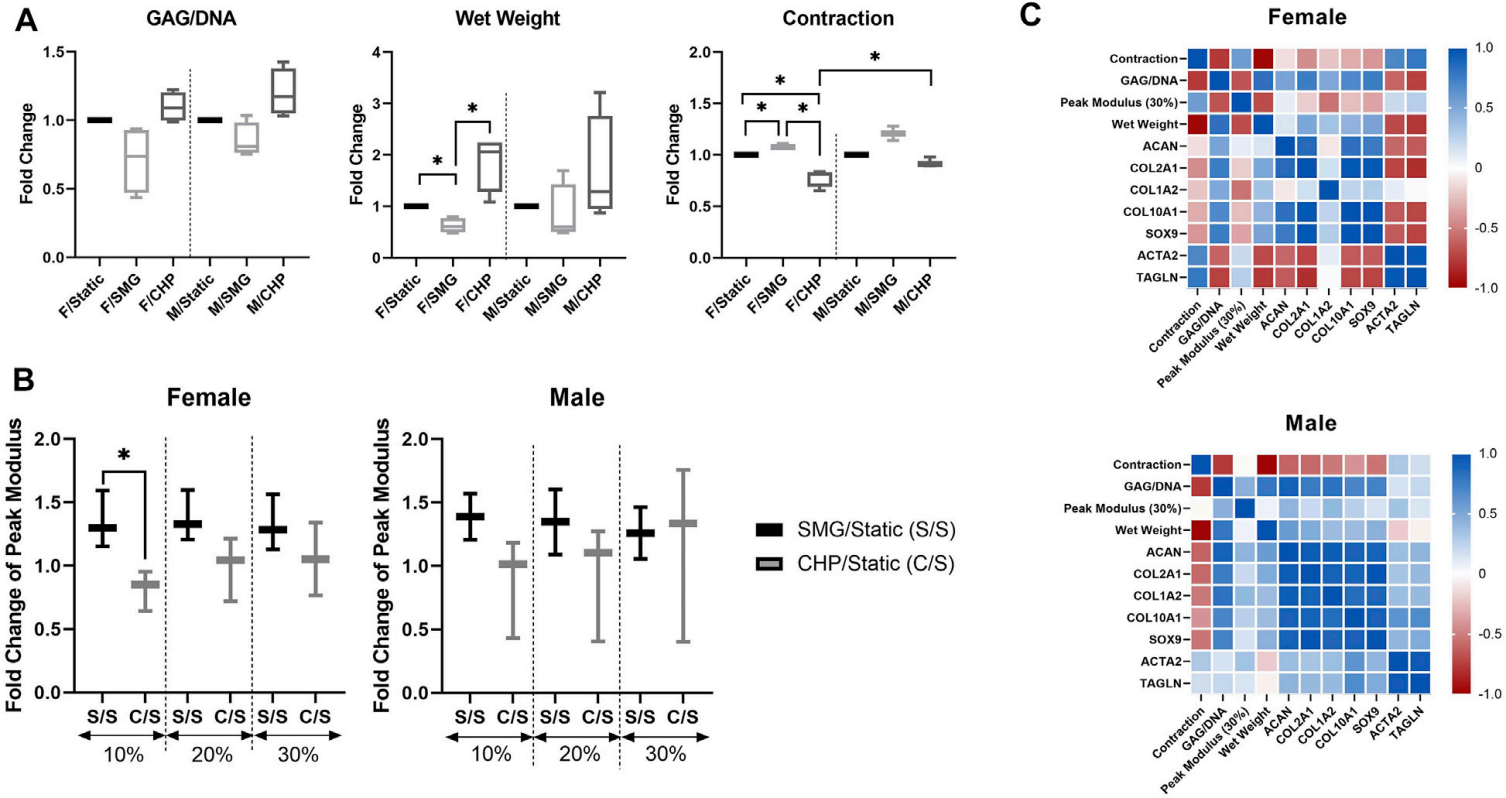
Effect of CHP and SMG on chondrogenesis related factors of female and male donors **(A)** Biochemical and morphological analysis; contraction is calculated as the % of area lost as compared to the original area. No statistical analysis was conducted for the contraction data with the male SMG group due to limited data points **(B)** Mechanical property analysis. No statistical analysis was conducted for the mechanical property data with the male SMG group due to limited data points **(C)** Pearson correlation heatmap of analyzed factors. Fold change value of characterized factors was calculated by normalizing the CHP or SMG group to its corresponding static group. Heatmap was generated by calculating the pairwise Pearson correlation coefficient of included factors. *Represents *p* < 0.05.

Sex-dependent differences were also assessed in other related aspects (Figure 4A). Quantitative GAG/DNA measurements showed that CHP increased while SMG decreased the GAG production per MFC. The tissue wet weights showed similar trends as the GAG/DNA ratios; CHP tissues weighed more, and SMG tissues weighed less on average than the static control groups. This difference in tissue wet weight was significant in only the female cohort between treatments. Further, at the end of the mechanical stimulation period, tissue constructs from all three experimental groups contracted to certain degrees. The percentage of contraction (percentage reduction in area) was quantified, and comparison among groups showed that SMG tissues had increased contraction compared to the CHP tissues. The contraction plot is presented as the percentage contracted in Supplementary Figure S4.

The differences among stimulation groups were significant within the female and male cohorts while also significant between female CHP and male CHP groups. For the mechanical properties of tissue constructs, SMG groups showed higher peak modulus at all tested strain levels for both sexes as expected based on contraction results, and the differences of the average fold-change decreased with increasing strain level. No significant differences were observed between female and male cohorts (Figure 4B).

To better understand the relationship among all factors of interest and the sex-dependent differences, a Pearson correlation network (Figure 4C) was generated for both the female and male cohorts with data from all three treatment groups. Some observations mentioned above were further confirmed with correlation coefficients, such as the positive correlation between GAG/DNA and wet weight, the negative correlation between GAG/DNA and contraction, and the positive correlation between *SOX9* and *COL2A1* expression levels. Overall, the correlation analysis yielded different patterns between the female and male cohorts, especially for two examined genes, *ACTA2* and *TAGLN,* that are characteristic of the contractile phenotype in dedifferentiated articular chondrocytes compared to the other factors (Parreno et al., 2017). The level of contraction was positively correlated with *ACTA2* and *TAGLN* expression levels in both female and male groups, confirming their indication for contraction levels. However, the female cohort showed differently a mostly strong negative correlation between the contractile genes and the rest of the factors compared to the male cohort. *ACTA2* and *TAGLN* activity is associated with cytoskeletal composition and structure (Killion et al., 2017; Tang and Gerlach, 2017), and thus results may suggest sex-dependent differences of cytoskeletal activity in response to mechanical stimulation.

### Comparison of Female and Male Transcriptome Response to CHP and SMG

**FIGURE 5:**
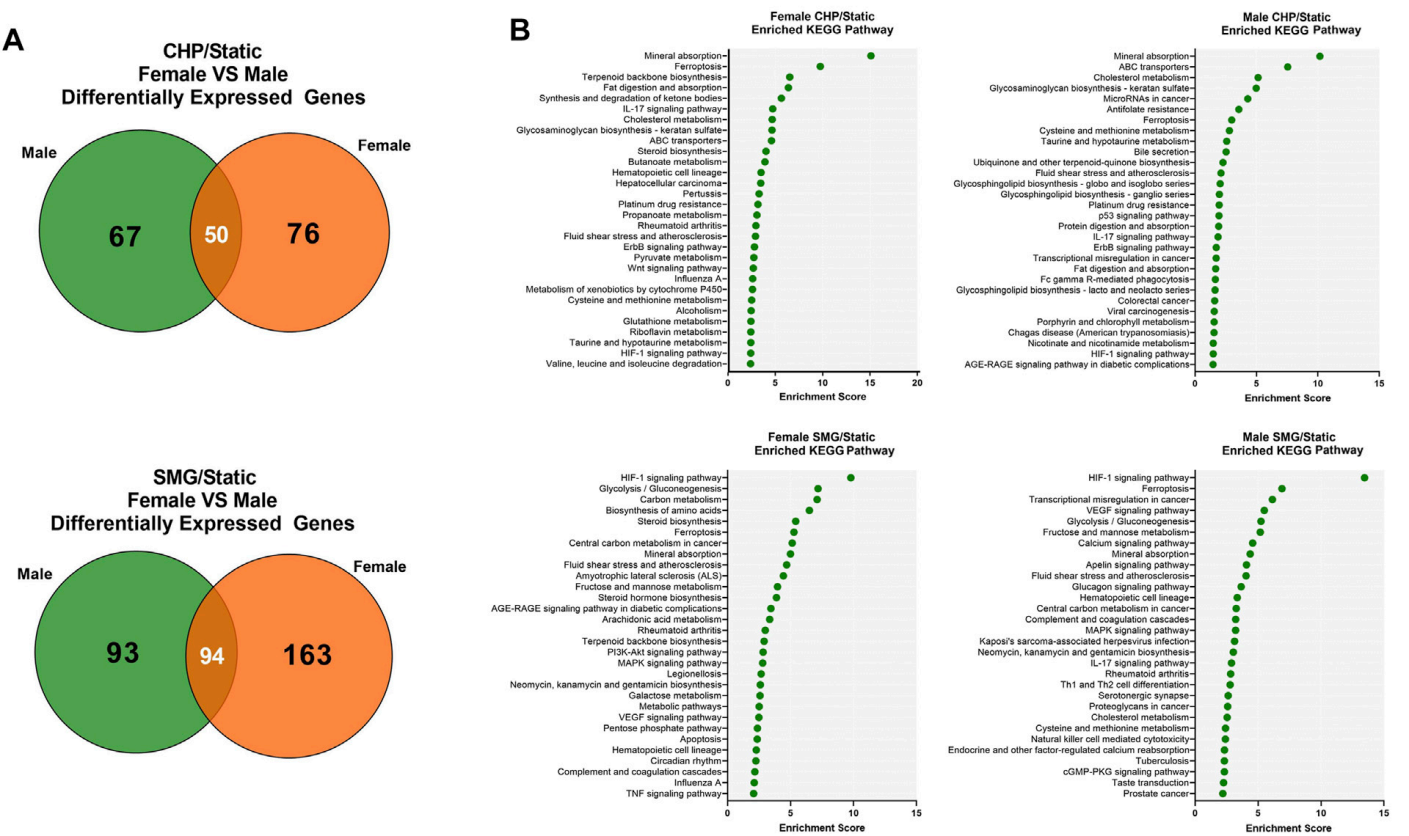
Transcriptome response of female and male donors to CHP and SMG. **(A)** Overlap of DEGs of female and male donors exposed to CHP and SMG. **(B)** Top enriched KEGG pathways by identified DEGs. KEGG terms were selected by *p*-value and plotted to the enrichment score.

Finally, we investigated the sex-dependent difference in the global transcriptome profile in response to mechanical loading and unloading. Consistent with the DEGs analysis for all donors combined, CHP and SMG uniquely regulated a large proportion of genes within each sex group (Figure 5A). We identified the top 25 enriched KEGG pathways for female and male tissues under CHP and SMG using the corresponding DEG sets (Figure 5B). For CHP, the most enriched KEGG pathway for both female and male groups was “mineral absorption.” Other relevant terms, such as “IL-17 signalling pathway”, “HIF-1 signalling pathway”, and “glycosaminoglycan biosynthesis,” were also observed for both sex groups. Although some of the enriched pathways were shared, a distinct gene profile with different magnitudes and sometimes the direction of modulation was observed for each sex group (Table 3). For example, the *NOTUM* gene in the Wnt-signalling pathway was significantly upregulated by 6.7-fold in CHP for the female cohort and only 1.8-fold in CHP for the male cohort. A large proportion of the top 25 KEGG pathways enriched by SMG overlapped with the pathways in CHP, but the corresponding gene profiles were different. For example, SMG did not significantly regulate the *NOTUM*, and it showed the opposite direction between female and male cohorts. The *MMP3* gene was downregulated by SMG but upregulated by CHP. Interestingly, in addition to the OA-related KEGG pathways, the “fluid shear stress and atherosclerosis” pathway was enriched for both female and male cohorts, suggesting an effect of mechanical stimulation from CHP and SMG on engineered meniscus constructs.

**TABLE 3:** Key enriched KEGG pathways and associated differentially expressed genes (DEGs) in CHP and SMG groups as compared to static controls within female and male donor cohorts. *q < 0.05, **q < 0.01, ***q < 0.001 represent statistical difference of the two groups in each fold change value.

*Gene*	Description	Fold change: CHP/Static			Fold change: SMG/Static		
		Female	Male	Combined	Female	Male	Combined
KEGG: Mineral absorption							
*FTH1*	Ferritin Heavy Chain 1	2.18*	3.47*	1.70*	3.47*	2.32*	2.77***
*HMOX1*	Heme oxygenase 1	2.94*	3.51**	2.83***	3.51**	3.28**	3.38***
*MT1E*	Metallothionein 1E	2.79**	1.96*	2.48***	1.96*	−1.09	1.32
*MT1G*	Metallothionein 1G	8.25*	3.29	9.38**	3.29	1.11	2.21
*MT1M*	Metallothionein 1M	2.59**	1.89*	2.20***	1.89*	−1.35	1.14
*MT2A*	Metallothionein 2A	2.59*	1.57	2.79***	1.57	1.18	1.35
*SLC30A1*	Solute Carrier Family 30 Member 1	2.21**	2.03*	1.70**	2.03*	1.23	1.57**
*SLC8A1*	Solute Carrier Family 8 Member A1	−2.13	1.15*	−1.44	1.15*	−3.13*	−2.91**
KEGG: Wnt signaling pathway							
*FOSL1*	FOS Like 1, AP-1 Transcription Factor Subunit	2.99*	3.88**	3.44***	1.16	1.88	1.53
*FZD2*	Frizzled Class Receptor 2	−2.22*	−1.25	−1.68*	−1.48	−1.12	−1.30
*NOTUM*	Notum, Palmitoleoyl-Protein carboxylesterase	6.71*	1.77	2.97*	1.17	−1.94	−1.48
*SFRP2*	Secreted Frizzled Related Protein 2	−6.22*	−3.03*	−4.07**	−2.55	−3.15	−2.82*
KEGG: HIF-1 signaling pathway							
*EGLN3*	Egl-9 Family Hypoxia Inducible Factor 3	1.00	2.49	1.58	−4.24*	−5.75*	−4.72***
*EN O 2*	Enolase 2	−1.24	1.48	1.11	−2.96*	−2.51	−2.74**
*GAPDH*	Glyceraldehyde-3-Phosphate dehydrogenase	−1.14	1.24	1.06	−2.27*	−2.21	−2.24**
*HK2*	Hexokinase 2	-1.61	1.03	−1.21	−2.20*	−2.66*	−2.41**
*HMOX1*	Heme oxygenase 1	2.94*	2.74*	2.83***	3.51**	3.28**	3.38***
*LDHA*	Lactate dehydrogenase A	1.21	1.31	1.26	−1.94*	−2.04*	−1.98***
*PDK1*	Pyruvate dehydrogenase kinase 1	−1.28	−1.13	−1.20	−2.89**	−3.43**	−3.11***
*PFKFB3*	6-Phosphofructo-2-Kinase/Fructose-2,6-Biphosphatase 3	−1.80	−1.15	−1.44	−2.33*	−2.34*	−2.33**
*PGK1*	Phosphoglycerate kinase 1	1.17	1.23	1.20	−2.04*	−2.24*	−2.13***
*TFRC*	Transferrin Receptor	2.80**	1.36	1.92**	2.55**	2.22*	2.35***
*VEGFA*	Vascular Endothelial Growth Factor A	−1.57	−1.11	−1.33	−5.48**	-5.95*	−5.68***
KEGG: IL-17 signaling pathway							
*CCL2*	C-C Motif Chemokine Ligand 2	3.85*	3.48**	3.62***	3.51**	1.41	2.17**
*FOSB*	FosB Proto-Oncogene, AP-1 Transcription Factor Subunit	137.8*	9.70	49.48**	2.82	3.66	3.40
*FOSL1*	FOS Like 1, AP-1 Transcription Factor Subunit	2.99*	3.88**	3.44***	1.16	1.88	1.53
*MAPK13*	Mitogen-Activated Protein kinase 13	−1.51	1.31	−1.07	−3.05*	−3.01*	−3.03***
*MMP13*	Matrix metallopeptidase 13	1.56*	1.38	1.51	1.64	2.57*	1.89**
*MMP3*	Matrix metallopeptidase 3	10.5**	4.36	6.47***	−1.80	−3.99*	−2.82**
KEGG: Fluid Shear stress and atherosclerosis							
*CCL2*	C-C Motif Chemokine Ligand 2	3.85*	3.48**	3.62***	3.51**	1.41	2.17**
*HMOX1*	Heme oxygenase 1	2.94**	2.74**	2.83***	3.51**	3.28**	3.38***
*MAP3K5*	Mitogen-Activated Protein kinase kinase 5	−1.37	−1.39	−1.38	2.38*	1.49	1.87**
*MAPK13*	Mitogen-Activated Protein kinase 13	−1.51	1.31	−1.07	−3.05*	−3.01*	−3.03***
*MGST1*	Microsomal Glutathione S-Transferase 1	2.21**	1.40	1.65**	1.86*	1.25	1.43**
*NQ O 1*	NAD(P)H Quinone dehydrogenase 1	3.83***	2.40**	2.88***	4.04***	2.42**	2.97***
*PLAT*	Plasminogen Activator, Tissue Type	1.51	2.27	1.88*	2.51*	2.43*	2.47**
*PRKAA2*	Protein kinase AMP-Activated Catalytic Subunit Alpha 2	−1.37	1.44	1.03	−4.63*	-2.90	−3.70**
*VEGFA*	Vascular Endothelial Growth Factor A	−1.57	−1.11	−1.33	−5.48**	−5.95*	−5.68***

In relation to mechanobiology of the meniscus, we investigated the transcriptome profile changes of various mechanosensitive molecules such as *TRPV1/4*, *PIEZO1*, *TMEM63A/B/C*, and *RUNX2* (McNulty and Guilak, 2015; Servin-Vences et al., 2017; Agarwal et al., 2021; Hwang et al., 2021; Lee et al., 2021). Furthermore, the mechanotransduction function of *FOSB* in MFCs (Szojka et al., 2021a) was reported by Szojka et al., and its function in the IL-17 signalling pathway was well demonstrated (Benderdour et al., 2002). Finally, Vyhlidal et al. recognized the potential of caveolae molecules such as *CAV1/2* in the mechanotransductive mechanism of the meniscus, but this needs to be further verified in future studies (Vyhlidal and Adesida, 2021). The transcriptome changes are summarized in Table 4. Within our dataset, only *CAV1*, *CAV2*, and *FOSB* are significant by *q*-value for loading regime comparison and sex-based comparison. *CAV2* was regulated in the opposite direction by CHP and SMG. CHP upregulated *FOSB* by 49.5-fold compared to 3.4-fold by SMG. The difference was even larger in female tissues (137.8-fold by CHP and 2.8-fold by SMG), and in comparison, the fold change of *FOSB* expression was similar by CHP and SMG for male tissues (9.7-fold and 3.7-fold). Other mechanosensitive molecules were not significantly modulated from the treatments. The transcriptome profile changes suggest sex-dependent differences in the mechanotransduction mechanisms as well as varying capabilities to sense cytoskeletal structural changes.

**TABLE 4:** Relevant mechanosensitive molecules in CHP and SMG groups as compared to static controls within female and male donor cohorts. *q < 0.05, **q < 0.01, ***q < 0.001 represent statistical difference of the two groups in each fold change value.

*Gene*	Description	Fold change: CHP/Static			Fold change: SMG/Static		
		Female	Male	Combined	Female	Male	Combined
*CAV1*	Caveolin 1	1.92*	1.51	1.68**	1.40	1.40	1.40*
*CAV2*	Caveolin 2	1.42*	1.28	1.34**	1.07	−1.15	−1.04
*FOSB*	FosB Proto-Oncogene, AP-1 Transcription Factor Subunit	137.8*	9.70	49.48**	2.82	3.66	3.40
*PIEZO1*	Piezo Type Mechanosensitive Ion Channel Component 1	1.19	1.41	1.30	-1.02	1.09	1.04
*RUNX2*	RUNX Family Transcription Factor 2	1.13	−1.13	1.02	−1.14	1.07	−1.04
*TMEM63A*	Transmembrane Protein 63A	−1.05	−1.13	−1.09	1.15	1.22	1.19
*TMEM63B*	Transmembrane Protein 63B	1.04	1.18	1.11	1.25	1.38	1.32
*TMEM63C*	Transmembrane Protein 63C	1.43	1.42	1.43	−1.24	−1.20	−1.22
*TRPV1*	Transient Receptor Potential Cation Channel Subfamily V Member 1	−1.62	−1.21	−1.37	−1.16	−1.03	−1.08
*TRPV4*	Transient Receptor Potential Cation Channel Subfamily V Member 4	1.25	1.02	1.11	−1.10	−1.16	−1.13

## Discussion

Currently, there is no existing model to reflect the physiological mechanism of OA holistically, but several *in-vitro* and *in-vivo* models have been developed to answer questions regarding the mechanisms of OA (Cope et al., 2019). This study aimed to evaluate the effects of mechanical loading and unloading *via* CHP and SMG, respectively, on the OA-related profile changes of engineered meniscus tissues and explore biological sex-related differences. This can serve as an *in-vitro* model to investigate the cellular and molecular profiles responsible for the sex-dependent incidence of OA disease.

Cartilage is a highly mechanosensitive tissue, and appropriate levels of mechanical stimulation are crucial for homeostasis and healthy cartilage development. Mechanical stimuli are transmitted by the pericellular matrix (PCM) (Melrose et al., 2006; Youn et al., 2006) to the chondrocyte surface and sensed by mechano-receptors, triggering a cascade of downstream activities (Zhao et al., 2020). The importance of mechanical loading under normal gravity environments has been demonstrated by studies examining the protective effects of moderate loading against tissue degradation as well as investigating the unwanted consequences from unloading. From several *in-vitro* and *in-vivo* models, mechanical loading has been shown to attenuate inflammatory cytokine-induced expression of matrix-degrading enzyme (Ohtsuki et al., 2019), upregulating the content of sulphated glycosaminoglycan (sGAG), aggrecan, cartilage oligomeric matrix protein, type II collagen, and lubricin (PRG4) (Antunes et al., 2020), and modulating relevant pathways such as the HIF-1 (Huang et al., 2017) and IL-4 (He et al., 2019) signalling pathways. On the contrary, prolonged mechanical unloading by space flight has been shown to accelerate cartilage degeneration (Herranz et al., 2013; Fitzgerald, 2017; Fitzgerald et al., 2019). Although several studies suggested that SMG was beneficial for preserving a chondrogenic phenotype through induction of 3D aggregates from monolayer cultures (Aleshcheva et al., 2015; Wuest et al., 2018; Wehland et al., 2020) and promoted cartilaginous components deposition for scaffold cultures (Freed and Vunjak-Novakovic, 1997), the degree of hypertrophic differentiation was not investigated in these studies. Through regulation of key genes and molecular pathways, SMG was also reported to increase activities associated with cartilage catabolism (Stamenković et al., 2010; Wang et al., 2010; Laws et al., 2016) and promoted hypertrophic differentiation of chondrocytes (Jin et al., 2013; Weiss et al., 2017).

In addition to cartilage, the meniscus also plays a critical role in the biomechanics of the knee joint. Evidence has shown that the role of meniscus fibrochondrocytes (MFCs) in response to mechanical signals affects the physiological, pathological, and repair response of the meniscus (McNulty and Guilak, 2015). The PCM of MFCs is also involved in mechanotransduction, although it may play a protective role against larger stresses and strains (Gupta and Haut Donahue, 2006). Various *in-vivo* studies have documented that mechanical stimulation can drive both anti- and pro-inflammatory responses in MFCs (Ferretti et al., 2005; Killian et al., 2014), as well as the detrimental effects of mechanical unloading from joint immobilization on meniscus development, function, and repair (Videman et al., 1979; Klein et al., 1989; Anderson et al., 1993; Djurasovic et al., 1998; Mikic et al., 2000; Bray et al., 2001). Additionally, several *in-vitro* studies have shown the anabolic effect of mechanical loading on MFCs with enhanced meniscus ECM components like *ACAN* expression and collagens and mechanical properties (Aufderheide and Athanasiou, 2006; Puetzer et al., 2012). Finally, there have been various *in-vitro* meniscus repair models that utilized mechanical loading to suppress IL-1 mediated increases in MMP activity, enhance sGAG production, and increase integrative strength of the engineered tissue constructs (McNulty et al., 2007; McNulty and Guilak, 2008; McNulty et al., 2010; Riera et al., 2011; McNulty and Guilak, 2015).

Consistent with these previous reports, our results showed that mechanical loading *via* CHP increased the deposition of type II collagen and aggrecan, supported by immunofluorescence staining and gene expression analysis. In addition, the Safranin-O staining and biochemical quantification of GAG production per cell confirmed that CHP increased the chondrogenic potential of meniscus MFCs. As expected, the wet weight of engineered constructs in the CHP group was the highest among the three treatment groups since the GAG is a major water-binding component of cartilage (KIANI et al., 2002). The ratio of *COL2A1* to *COL1A2* has also been used to evaluate chondrogenic capacity (Wuest et al., 2018; Wehland et al., 2020), and in our results, CHP substantially increased the *COL2A1*/*COL1A2* ratio compared to SMG groups. Although upregulation of type II collagen deposition and *COL2A1* expression was observed in the SMG groups compared to static controls, the magnitude of upregulation was not less than in the CHP groups. The GAG/DNA ratio of the SMG group was also lower than static controls in addition to a stronger intensity of type X collagen staining. Taken together, our data highlights the detrimental effect of SMG on the chondrogenic capacity of the engineered meniscus and suggests that mechanical unloading increases the hypertrophic differentiation of MFCs, driving them to display OA-like characteristics.

To the best of our knowledge, this is one of the first studies to investigate the effect of mechanical loading and unloading on cartilage models by examining global transcriptome profile alterations. The ECM of chondrocytes is crucial for regulating key functions through receptor-mediated matrix-cell interactions. The composition of the ECM and the bound signalling molecules largely influences the chondrogenic capacity of embedded chondrocytes (Gao et al., 20142014). By overlaying the DEGs, we found that CHP and SMG regulated MFC functions through largely different mechanisms. As listed in Table 2, several Wnt-signaling pathway-related genes were strongly regulated by CHP, in addition to various matrix remodelling enzymes. But for SMG, the genes with the highest fold-change functioned in more general ways, such as the several growth factors encoding genes identified to regulate general development processes. The KEGG pathway analysis also showed that several chondrogenesis- and OA-related pathways such as mineral absorption, Wnt-signalling pathway, HIF-1 signalling pathways, and IL-17 signalling pathway were enriched by both CHP and SMG. However, the expression profile of related genes in terms of magnitude and direction was quite different (Table 3). It is worth mentioning that no sex hormone-related pathways were present in the top enriched pathways. Thus, the observed sex-dependent differences are independent of differences between sex-related hormones. Taken together, the comparative transcriptome analysis suggested a distinct effect of CHP and SMG on regulating chondrogenesis, but further investigations are needed to determine the specific underlying mechanisms.

The cellular and molecular mechanisms behind the well-documented sex discrepancy in OA incident rates are poorly understood. Many factors are believed to contribute to the higher incidence and severity of OA in females, such as age, psychosocial status, metabolic variables, hormonal differences, anatomical variations, and inflammatory disease (Ferre et al., 2019). While the focus of many previous studies was mainly on bone shape (Yang et al., 2014; Wise et al., 2016; Frysz et al., 2020), gait kinematics (Phinyomark et al., 2016; Ro et al., 2017; Allison et al., 2018), and sex hormones (Zazulak et al., 2006; Brennan et al., 2010; Boyan et al., 2013b; Wang et al., 2013; Antony et al., 2016), little effort was invested into determining differences in the global transcriptome profile. Two studies reported that female OA patients have higher levels of inflammatory cytokines in the synovial fluid than males (Hooshmand et al., 2015; Kosek et al., 2018), but the underlying signalling mechanism was not investigated. To explore the sex-dependent differences in OA pathogenesis, we separated our donor cohort based on sex and compared the OA-related characteristics.

Although the female and male donor cohorts included in this study showed highly variable trends in terms of histological and immunofluorescence staining for cartilage markers like aggrecan and type II collagen, an expected similar trend was observed for *COL2A1*, *SOX9*, *ACAN*, and *COL2A1/COL1A2* average fold-change levels across donor and treatment groups. The average fold change level for *COL10A1* was comparable between the CHP and SMG groups for both sexes. However, one female donor showed significantly higher fold-changes for *COL10A1* while the remaining female cohort showed a generally reduced expression of *COL10A1* in CHP compared to SMG, but not significant. The observed deposition of type X collagen in immunofluorescence staining shows high variability, and sex-dependent differences are difficult to elucidate. Our results suggest overall that at the protein level, the effect of mechanical stimulation is more dominant than sex differences for the deposition of cartilaginous components.

A significant sex-dependent difference was observed for tissue contraction between female and male CHP groups. Additionally, the Pearson correlation network generated for the female and male donor cohorts shows clear sex-dependent differences in expression trends between contractile genes *ACTA2* and *TAGLN* in relation to the other factors investigated in this study. Firstly, the measured contraction based on the percentage of reduction in tissue area positively correlated with *ACTA2* and *TAGLN* expression levels in both female and male cohorts. This suggests that these contractile genes are indeed correlated with physical contraction levels in our study. Interestingly, *ACTA2* and *TAGLN* expression levels in relation to other factors show distinct sex-dependent differences. Among the female cohort, the contractile genes showed a mostly strong negative correlation with the other factors, while the genes in the male cohort showed a generally positive correlation. Mechanical loading has been suggested to mediate the function of chondrocytes by stimulating the reorganization of cytoskeleton (Killion et al., 2017; Tang and Gerlach, 2017). And simulated microgravity was also reported to alter the structure of cytoskeleton components (Wehland et al., 2020) and regulate the expression of several cytoskeletal genes (Aleshcheva et al., 2015). Since *ACTA2* and *TAGLN* activity is associated with cytoskeletal composition and structure, the opposite trend in correlation with other factors between the female and male cohort may suggest sex-dependent differences from cytoskeletal activity in response to varying levels of mechanical stimulation. Furthermore, the mechano-sensitive gene *FOSB* in the IL-17 signalling pathway was identified in our dataset to show a 137.8-fold upregulation from CHP in the female cohort while only observing a 9.7-fold increase in the male cohort. This may suggest sex-dependent differences in the mechanotransduction mechanisms as well as varying capabilities to sense cytoskeletal structural changes arising from mechanical stimulation.

There are several limitations to our model explored in this study. Firstly, we did not have any tissue samples from OA patients to confirm the OA-phenotype observed in our model. However, the modulation of several OA markers (Kiraly et al., 2017) from SMG such as the upregulation of MMP13 and the increased staining of collagen type X, reasonably suggest that the SMG treatment is pushing the engineered tissues constructs towards an OA-phenotype, and this effect is likely to increase with longer treatment periods. Another limitation is that even prolonged joint mobility under normal gravity can induce an OA-phenotype, and thus the static group in this study may also serve as an alternate condition for simulating OA. However, the static group here is meant as a baseline control between the two mechanical treatments and serves to reduce donor-to-donor variability by normalizing measurements to the static control within each donor. Finally, a limitation in our model is that although the engineered meniscus constructs contain the necessary component of fibrocartilage, the tissue microenvironment experienced by the cells is likely different from that of the native meniscus. In particular, the cells in the native meniscus may respond differently to receptor-mediated cell-ECM interactions from mechanical load than in the engineered meniscus models, especially due to differences in the matrix stiffness. We hope to address this limitation in our future studies by evaluating cytoskeleton factors and measurements and using substrates with tunable stiffnesses such as hydrogels.

## Conclusion

Taken together, our data suggest that engineered meniscus tissues responded to mechanical loading and unloading *via* CHP and SMG in a sex-dependent manner. Mechanical unloading *via* SMG was shown to induce an OA-like profile, while mechanical loading *via* CHP promotes elements of chondrogenesis. Within each mechanical stimulation group, female and male donor cohorts show sex-dependent differences in the magnitude and direction of many differentially expressed genes, as well as tissue contraction and correlation of contractile genes with the other factors investigated in this study. The combination of CHP and SMG can feasibly serve as an *in-vitro* model to study the cellular and molecular mechanisms of KOA and provide a platform for exploring potential drug-targetable pathways as therapeutics.

## Data Availability

The datasets presented in this study can be found in online repositories. The names of the repository/repositories and accession number(s) can be found below: GEO and GSE192982.
