# Supplementary material for: Engineered Human Meniscus in Modeling Sex Differences of Knee Osteoarthritis in Vitro
Source: Front Bioeng Biotechnol. 2022 Feb 15;10:823679. doi: 10.3389/fbioe.2022.823679 (PMC8904202; doi:10.3389/fbioe.2022.823679)
Supplement: Supplementary file 1 [file Table1.DOCX]

**Supplementary Table 1**. Real-time qPCR Primer Sequences

| **Gene** | **Forward** | **Reverse** | **GenBank Accession** |
| --- | --- | --- | --- |
| *ACAN* | AGGGCGAGTGGAATGATGTT | GGTGGCTGTGCCCTTTTTAC | NM_001135.3 |
| *Β-actin* | AAGCCACCCCACTTCTCTCTAA | AATGCTATCACCTCCCCTGTGT | NM_001101.4 |
| *B2M* | TGCTGTCTCCATGTTTGATGTATCT | TCTCTGCTCCCCACCTCTAAGT | NM_004048.3 |
| *COL1A2* | GCTACCCAACTTGCCTTCATG | GCAGTGGTAGGTGATGTTCTGAGA | NM_00008 9.3 |
| *COL2A1* | CTGCAAAATAAAATCTCGGTGTTCT | GGGCATTTGACTCACACCAGT | NM_001844.5 |
| *COL10A1* | GAAGTTATAATTTACACTGAGGGTTTCAAA | GAGGCACAGCTTAAAAGTTTTAAACA | NM_000493.3 |
| *SOX9* | CTTTGGTTTGTGTTCGTGTTTTG | AGAGAAAGAAAAAGGGAAAGGTAAGTTT | NM_000346.3 |
| *YWHAZ* | TCTGTCTTGTCACCAACCATTCTT | TCATGCGGCCTTTTTCCA | NM_003406.3 |
