# Supplementary material for: Engineered Human Meniscus in Modeling Sex Differences of Knee Osteoarthritis in Vitro
Source: Front Bioeng Biotechnol. 2022 Feb 15;10:823679. doi: 10.3389/fbioe.2022.823679 (PMC8904202; doi:10.3389/fbioe.2022.823679)
Supplement: Supplementary file 2 [file DataSheet1.PDF]

Supplementary Materials

1 Mechanical property assessment loading protocol

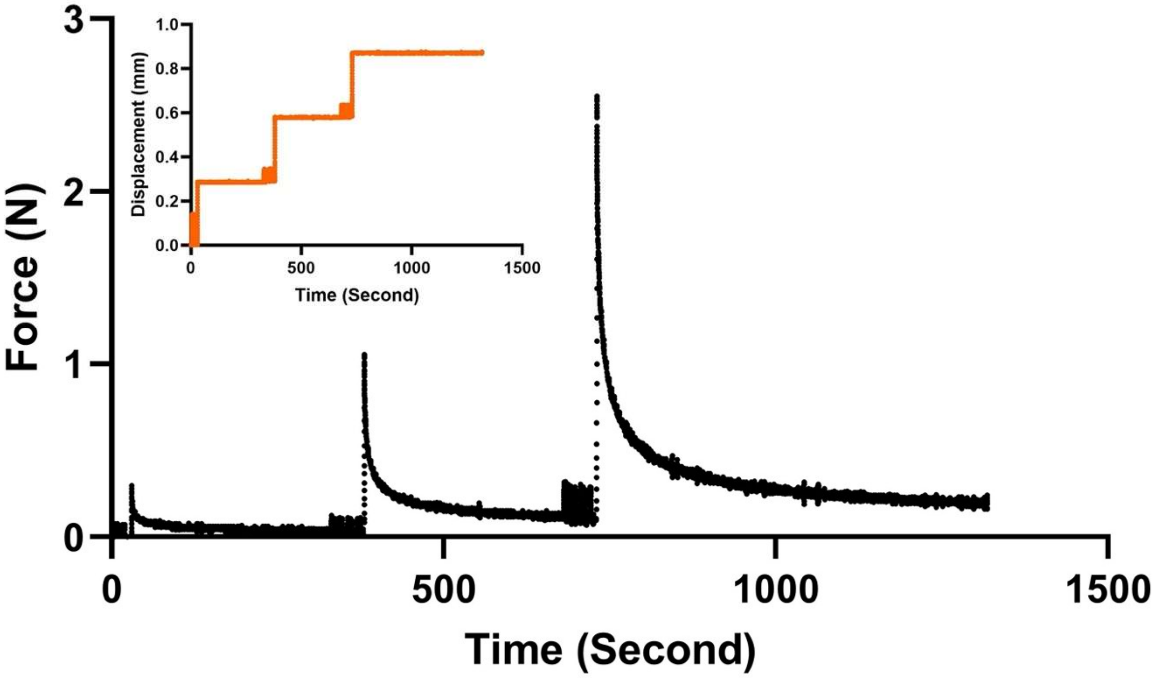

Supplementary Figure 1. Strain-controlled undefined compression test was used to measure the peak modulus of samples at three indicated strain levels.

2 Correlation between RT-qPCR and RNA-Seq data.

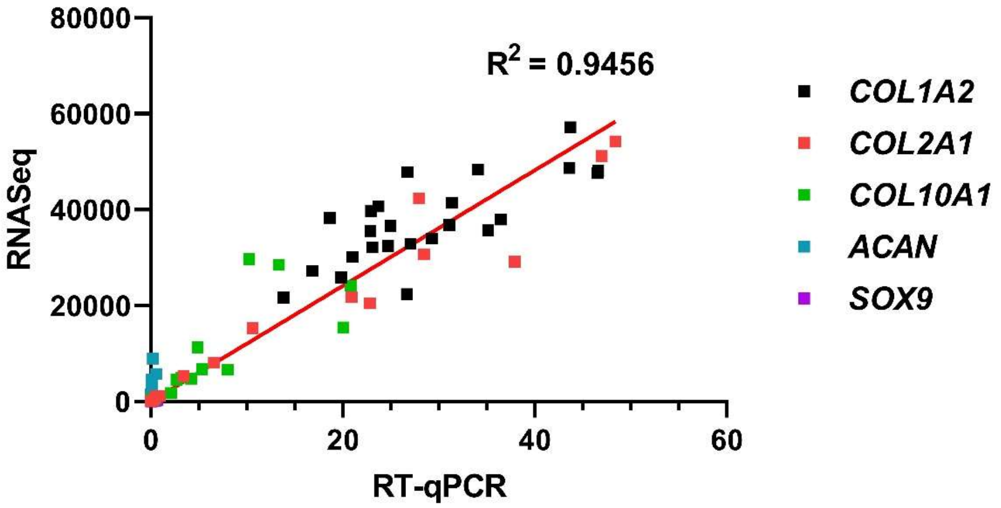

Supplementary Figure 2. Linear regression was used to evaluation to correlation between gene expression value measured by qRT-PCR and RNA-Seq.

3 Detailed sequence of mechanical testing protocol.

1. Dwell (wait): 5s
2. Sine (cyclic compression): -5% strain amplitude at 1 Hz for 15 cycles
3. Dwell: 10s
4. Ramp (increase compression): at a speed of -50% strain/s to -10% strain
5. Dwell: 300s
6. Sine: -2% strain amplitude at 0.5 Hz for 10 cycles
7. Dwell: 5s
8. Sine: -2% strain amplitude at 1 Hz for 10 cycles
9. Dwell: 5s
10. Sine: -2% strain amplitude at 2 Hz for 10 cycles
11. Dwell: 5s
12. Ramp: at a speed of -50% strain/s to -20% strain
13. Dwell: 300s
14. Sine: -2% strain amplitude at 0.5 Hz for 10 cycles
15. Dwell: 5s
16. Sine: -2% strain amplitude at 1 Hz for 10 cycles
17. Dwell: 5s
18. Sine: -2% strain amplitude at 2 Hz for 10 cycles
19. Dwell: 5s
20. Ramp: at a speed of -50% strain/s to -30% strain
21. Dwell: 600s
22. Sine: -2% strain amplitude at 0.5 Hz for 10 cycles
23. Dwell: 5s
24. Sine: -2% strain amplitude at 1 Hz for 10 cycles
25. Dwell: 5s
26. Sine: -2% strain amplitude at 2 Hz for 10 cycles
27. Dwell: 5s
28. Ramp: release compression

4 Tissue contraction presented as % contracted

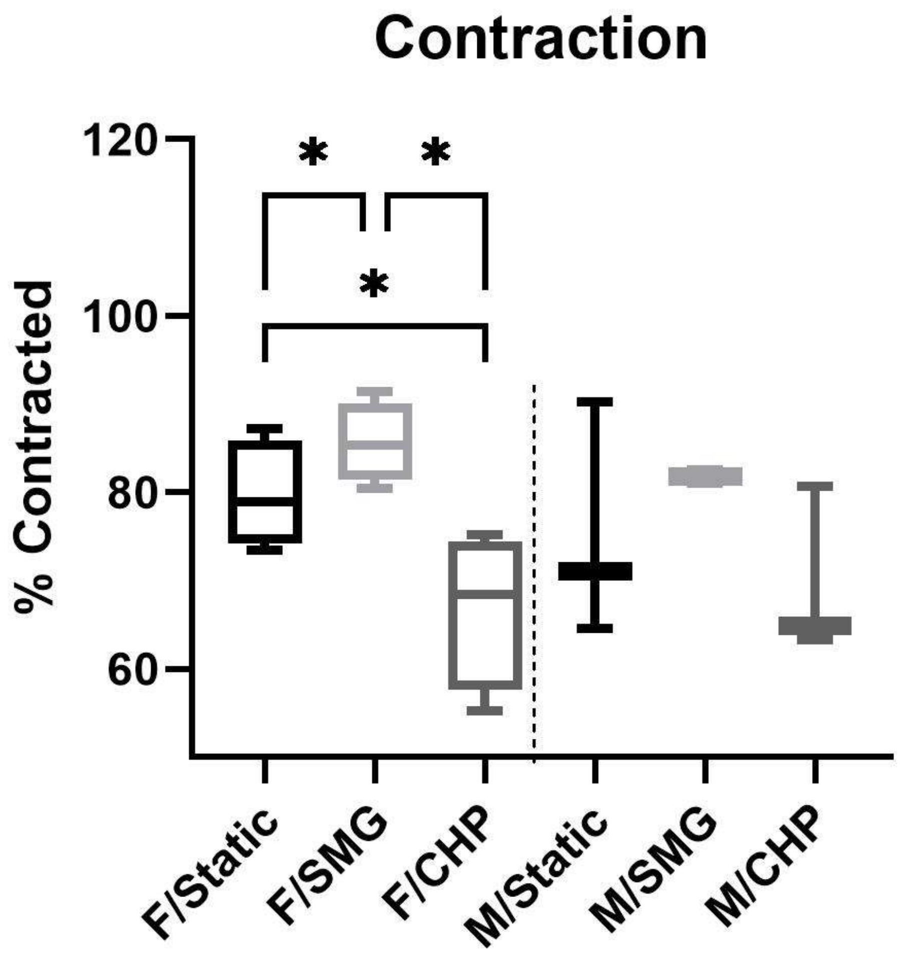

Supplementary Figure 4. Effect of CHP and SMG on tissue contraction; contraction is calculated as the % of area lost as compared to the original area. No statistical analysis was conducted for the contraction data with the male SMG group due to limited data points
